# Apparent diffusion coefficient in normal and abnormal pattern of intervertebral lumbar discs: initial experience^☆^

**DOI:** 10.1016/S1674-8301(11)60026-21

**Published:** 2011-05

**Authors:** Gang Niu, Xuewen Yu, Jian Yang, Rong Wang, Shaojuan Zhang, Youmin Guo

**Affiliations:** Department of Diagnostic Radiology, the First Hospital of Medical School, Xi'an Jiaotong University, Xi'an, Shaanxi 710061, China

**Keywords:** intervertebral lumbar disc, apparent diffusion coefficient, disc bulging, disc herniation

## Abstract

The aim of the present study was to compare the relationship of morphologically defined non-bulging/herniated, bulging and herniated intervertebral lumbar discs with quantitative apparent diffusion coefficient (ADC). Thirty-two healthy volunteers and 28 patients with back pain or sciatica were examined by MRI. All intervertebral lumbar discs from L1 to S1 were classified according to morphological abnormality and degenerated grades. The ADC values of nucleus pulposus (NP) were measured and recorded. The significant differences about mean ADC values of NP were found between non-bulging/herniated discs and bulging discs as well as herniated discs (*P* < 0.05), whereas there were no significant differences in ADC values between bulging and herniated discs (*P* > 0.05). Moreover, statistically significant relationship was found in the mean ADC values of NP between “non-bulging/herniated and non-degenerated discs” and “non-bulging/herniated degenerated discs” as well as herniated discs (*P* < 0.05). Linear regression analysis between ADC value and disc level revealed an inverse correlation (*r* = -0.18). The ADC map of the NP is a potentially useful tool for the quantitative assessment of componential and molecular alterations accompanied with lumbar disc abnormalities.

## INTRODUCTION

Disc bulging and herniation are common both in China and Western countries[1],[2]. Intervertebral disc degeneration (IVDD) is also progressive and age-related in adults. If exacerbated, it may cause disc herniation[3]. Magnetic resonance imaging (MRI) can reflect both the macromolecular concentrations and the structural integrity in the intervertebral disc (IVD), so it has been considered as a useful noninvasive method of estimating disc bulging, herniation and IVDD in humans and animals *in vivo*[4]–[6].

Diffusion-weighted MRI is a noninvasive method of measuring the diffusion of water within tissue *in vivo* and apparent diffusion coefficient (ADC) provides an estimate of free diffusion of unbound water; therefore, it could be also used as a quantitative tool to assess pathologic alterations in water diffusion of disc disease[7]. Previous studies showed strong evidence that decreased diffusion is associated with reduction in nutrient supply and measurement of ADC value in the nucleus pulposus (NP) could reflect the degenerative changes and integrity of disc matrix[8]–[12]. Apart from the analysis of disc degeneration, to the best of our knowledge, ADC map has not yet been evaluated in disc abnormalities such as bulging and herniation. The aim of this study was to compare the ADC values of NP between disc bulging, herniated and “non-bulging/herniated discs”.

## MATERIALS AND METHODS

### Study Population

The subjects were consecutively recruited from January 2009 to October 2009. The group included 32 healthy volunteers (16 men and 16 women) without symptoms of back pain or sciatica and the median age was 38 years (age range 18-73 years). Others included 28 patients (12 men and 16 women) with back pain or sciatica and the median age was 47 years (range 20-74 years). Individuals were excluded in the presence of diabetes mellitus, major systemic diseases, serious illnesses (e.g., tumor and infection), back surgery, spinal fractures, or diagnosed osteoporosis. This prospective study was approved by the Institutional Review Board in the First Hospital of Medical School, Xi'an Jiaotong University and all participants provided written informed consent before enrollment.

### MRI imaging

Lumbar spine imaging was achieved on a whole-body 1.5T clinical scanner (Gyroscan, release 9.1.2; Philips Medical Systems, the Netherlands). Each MR scanning in this study was performed in the afternoon in order to diminish possible influence on ADC and T_2_ value by diurnal variation in the intervertebral discs[13].

Conventional lumbar MRIs were acquired with a 4-channel cervical-thoracic-lumbar spinal (Syn-spine) coil by T_2_-weighted images (T_2_WI) fast spin echo (FSE) sequence in the sagittal and axial orientation. The scanning parameters were as follows: TR/TE, 3,500/1,200 ms; thickness, 4.0 mm; field of view (FOV), 250 mm; matrix, 512×512; number of signal acquisitions (NSA), 4; turbo factor, 17; number of sagittal sections, 11; number of axial sections in each disc, 3. Moreover, sagittal T1-weighted imaging was performed by FSE sequence with TR/TE, 400/14 ms; thickness, 4.0 mm; FOV, 256 mm; matrix, 512×512; NSA, 2; turbo factor, 3; number of sections, 11. The sagittal DWIs of the lumbar spine were obtained by a single-shot SE echo-planar imaging sequence (DWEPI) with Syn-spine coil in three orthogonal directions and then an average ADC was calculated from the three ADC directions. A b-value of 500 s/mm^2^ was chosen as a suitable compromise for diffusion-weighted imaging of intervertebral discs in this study. Other parameters were as follows: TR/TE of 3,000/81 ms, thickness of 6.0 mm, FOV of 250 mm, matrix of 512×512 and NSA of 8. The total scan time was 3 min 34 s for 3 slices passing through the lumbar center.

### Assessment of lumbar discs

**Fig. 1:**
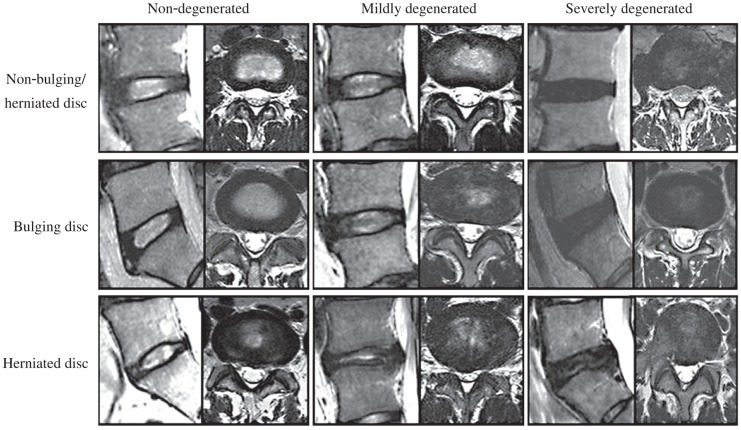
Different classifications of intervertebral disk. The rows show the classification of abnormality and the columns show classification of disc degerceration.

All discs were classified morphologically on MRI images by two experienced radiologists with over 10 years' experience in MR imaging of the spine. They divided all discs into three groups: non-bulging/herniation discs group, disc bulging group and disc herniation group (protrusion or extrusion) according to the degree of disc extension beyond the interspace (DEBIT). 1) Non-bulging/herniation discs indicates no disc extension beyond the DEBIT; 2) Bulge refers to circumferential, symmetric DEBIT (around the end plates); 3) Herniation includes two types: Protrusion indicates focal or asymmetric DEBIT into the canal, and the base against the parent disc broader than any other diameter of the protrusion. Extrusion means focal, obvious DEBIT, and the base against the parent disc narrower than any diameter of the extruding material itself, or no connection between displaced disc material and parent disc[14] (***Fig. 1***).

For evaluation of disc degeneration, radiologists also classified all discs into the following categories: non-degenerated, mildly-degenerated and severely-degenerated grades according to the absence or presence of three findings: 1) loss of disc height; 2) reduction in signal intensity on T_2_WI compared with that of normal intervertebral discs in the same individual; 3) loss of distinctness of the intranuclear cleft compared with that of normal discs in the same individual. Discs without three features were classified as non-degenerated discs. Moreover, discs with only one of these findings were defined as mildly degenerated discs, while those with at least two findings were defined as severely degenerated discs[11] (***Fig. 1***).

The two radiologists worked independently and without knowledge of the clinical information and findings on ADC map in three consecutive weeks. If disagreements occurred, they reviewed the images together and reached a consensus for analysis.

All discs were divided into three groups described above. The column displayed degenerative grades of discs according to three findings (loss of disc height; reduction in signal intensity or loss of distinctness of the intranuclear cleft on T_2_WI).

### Region of interest setting and measured

**Fig. 2:**
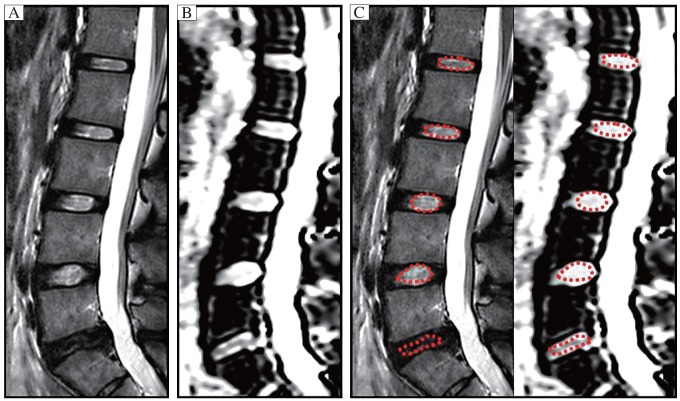
The elliptic ROIs with red dotted line were manually delineated in T_2_-weighted image (T_2_WI) at the center of lumbar disks to cover nucleus pulposus from L1 to L5. The irregular ROIs for L5-S1 were selected by the operator according to the expected location of inner portion of lumbar disks. Then these ROIs in T_2_WI were copied to the ADC maps, and ADC values measured. A: T_2_WI. B: ADC map. C: ROI setting.

All data were transferred into the imaging workstation (Easy Vision, Philips Medical Systems, USA). A radiologist performed the region of interest (ROI) in central discs from L1 to S1, including NP: 1) For non-degenerated and some of degenerated discs, an elliptical ROI, on the middle section of sagittal T_2_WI, was manually drawn in the inner portion of each lumbar disc with an area of 60-80 mm^2^, where it indicates the regions of NP; 2) For discs that the boundary between the NP and annulus fibrosus (AF) was not distinguishable, the irregular or regular ROIs were selected by the operator according to the expected location of the inner portion[15]. Then, the ROIs were copied to the ADC map at the same level, and the ADC values of NP were recorded for analysis (***Fig. 2***).

The typical sagittal T_2_WI with corresponding sagittal ADC map was acquired from a 30-year-old asymptomatic volunteer. The lumbar discs were graded according to the sagittal T_2_WI as follows: L1-L2, L2-L3 as mildly degenerated, L3-L4 and L4-L5 as non-degenerated and L5-S1 as severely degenerated. The elliptic ROIs with red dotted line were manually placed at the center of the lumbar discs including the NP. The irregular ROIs with red dotted line for L5-S1 were selected by the operator according to the expected location of the NP. Then, the ROIs were copied to the ADC map at the same level.

### Statistical analysis

The variations of ADC values of NP in lumbar intervertebral discs along with anatomic levels were assessed by Spearman correlation coefficient (*r*). The degree of correlation was evaluated by *r* absolute value as follows: *r* > 0.7 was defined as strong; 0.5 < *r* ≤ 0.7 as moderate; *r* ≤ 0.5 as weak. Statistical significance was set at *P* < 0.01. One-way ANOVA and post hoc Games-Howell tests were used for the comparison of the mean ADC values of NP in non-bulging/herniated discs, bulging and herniated discs. In addition, we also evaluated the relationship between the mean ADC values of NP and degenerative grades according to the three groups: non-bulging/herniated and non-degenerated group, non-bulging/herniated and degenerated group, and herniated group. A *P* value of less than 0.05 was considered significant. In this study, statistical analyses were performed by using SPSS for Windows version 13.0 (SPSS, Inc, IL, USA).

## RESULTS

**Fig. 3:**
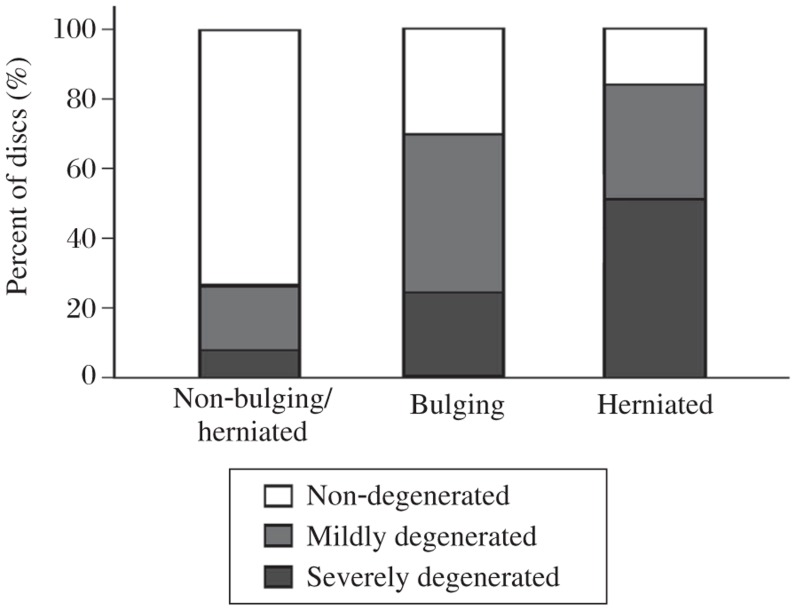
Comparison of the corresponding disc percentages of the different groups among non-bulging/herniated, bulging, and herniated discs.

In this study, a total of 300 intervertebral discs were categorized in the 60 participants (32 healthy volunteers and 28 patients). A total of 295 discs were successfully measured on ADC map. Five discs were excluded because three discs were obscured due to susceptibility artifacts at L3-S1 in one person, and two discs severely degenerated at L5-S1 had so narrow space that precluded the ROIs. The percentages of discs in the different classifications showed that most non-bulging/herniated discs were present in the non-degenerated disc group; nevertheless, most abnormal discs were seen in the mildly degenerated and severely degenerated groups (***Fig. 3***).

**Fig.4:**
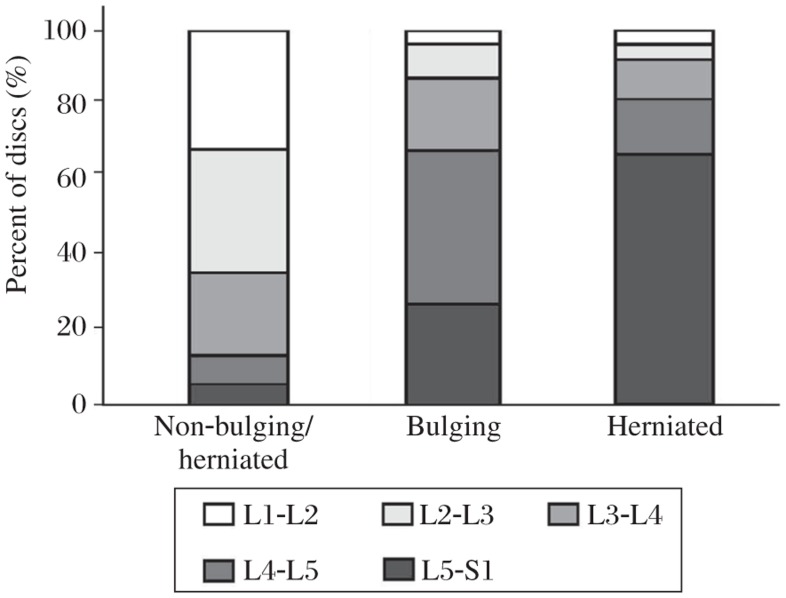
The bars for non-bulging/herniated, bulging and herniated discs represent the corresponding disc percentages of different disc levels.

**Fig.5:**
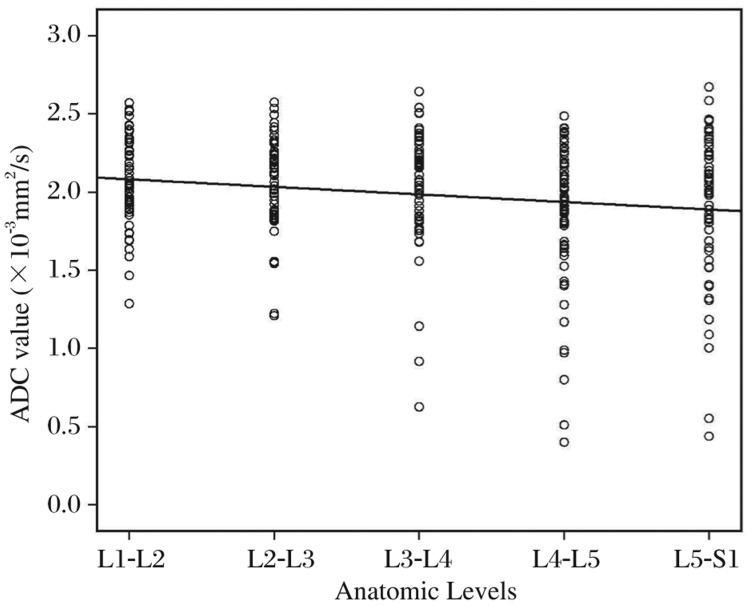
Lumbar disc level correlated with the apparent diffusion csefficient (ADC) values of the nucleus pulposus. ADC values for the nucleus pulposus region of all discs in each individual are illustrated. The solid line represents the regression line. *r* = -0.18, *P* = 0.002

Most of non-bulging/herniated discs were localized at the cephalad lumbar level, whereas most of abnormal discs were in the caudal lumbar position (***Fig. 4***). There was a weak inverse correlation between ADC value of NP and lumbar disc level by linear regression analysis ( *r* = -0.18, *P* < 0.01, ***Fig. 5***).

**Fig.6:**
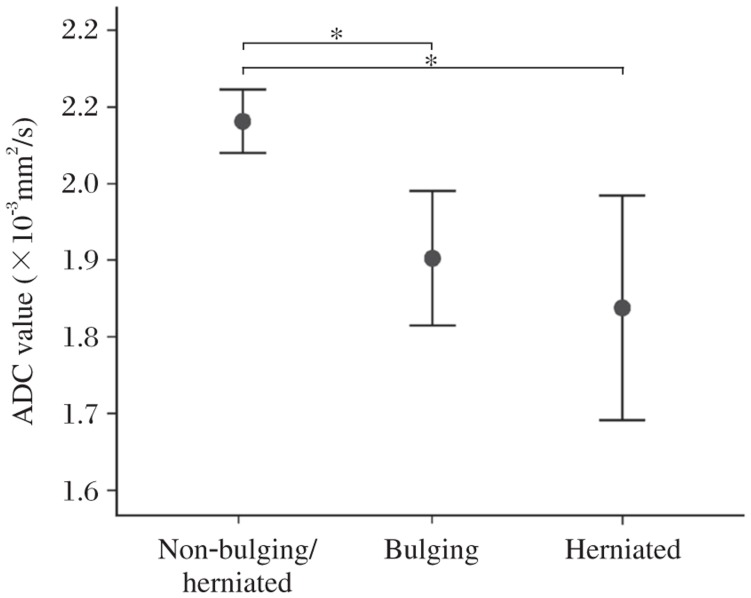
Comparison of the mean apparent diffusion coefficient (ADC) values of the nucleus pulposus (NP) among non-bulging/herniated, bulging, and herniated discs. The vertical bars represent the standard deviation from the mean with dark dots.*P < 0.05

The mean ADC values of NP for each group are (2.06±0.19)×10^−3^ mm^2^/s for non-bulging/herniated discs, (1.90±0.23)×10^−3^ mm^2^/s for bulging discs, and (1.83±0.32)×10^−3^ mm^2^/s for herniated discs, respectively. There was a significant relationship in the mean ADC values of NP between non-bulging/herniated discs and bulging discs, between non-bulging/herniated discs and herniated discs (*P* < 0.05, ***Fig. 6***), whereas there was no significant difference in the mean ADC values of NP between bulging and herniation (*P* > 0.05, ***Fig. 6***).

**Fig.7:**
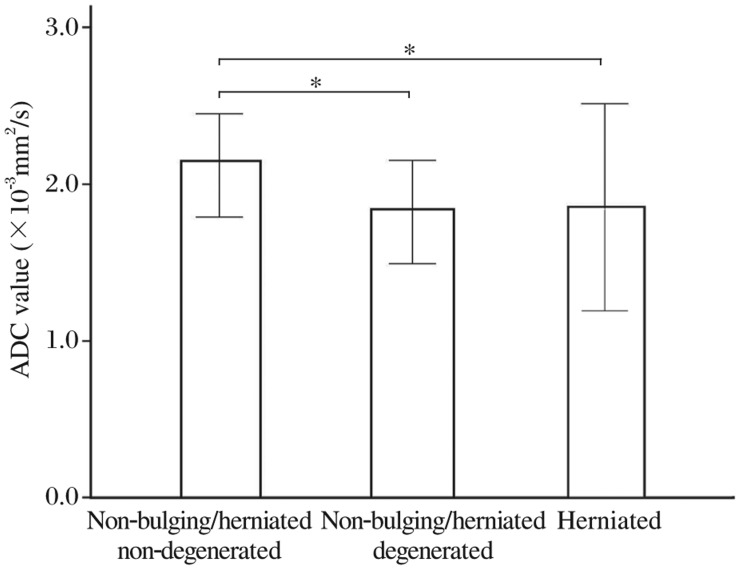
Relationship between apparent diffusion coefficient (ADC) values of the nucleus pulposus (NP) and different categorizations. The mean ADC value of NP in different lumbar disc categorizations is shown by clustered graph. The vertical bars represent the standard deviation, *P < 0.05.

The mean ADC values of NP in different categories are shown respectively: (2.12±0.23)×10^−3^ mm^2^/s for the non-bulging/herniated and non-degenerated group, (1.81±0.29)×10^−3^ mm^2^/s for the non-bulging/herniated and degenerated group, and (1.83±0.42)×10^−3^ mm^2^/s for herniated discs. There was a marked statistical difference in the mean ADC values of NP between the “non-bulging/herniated and non-degenerated group” and herniated group as well as the “non-bulging/herniated and degenerated group” (*P* < 0.05, ***Fig. 7***).

## DISCUSSION

In this study, the ADC values of NP were measured in subjects with different categorizations including disc abnormalities and disc degeneration. It confirmed the previous report that most non-bulging/herniated discs were at the cephaled level, and most of the bulging and herniated discs were localized at the caudal level[2] whereas there was a weak inverse correlation between the ADC values of NP and lumbar disc level (*r* = -0.18, *P* < 0.01). In addition, the ADC value of non-bulging/herniated discs was markedly higher than bulging and degenerated discs, and revealed statistically significant differences (*P* < 0.05). Further results depicted statistically significant differences in the ADC values of the NP between non-bulging/herniated and non-degenerated group and herniated group, between non-bulging/herniated and degenerated group and herniated group (*P* < 0.05).

The lumbar intervertebral disc is the largest avascular structure in the body. Nutrients are dependent on diffusion of fluid either from the vertebral bodies across cartilaginous endplate or through the AF from the surrounding capillaries to reach the NP, and metabolic waste products of discs are removed from the tissue by the reverse route[16],[17]. Healthy IVD supports axial loading via a pressurized NP. Changes in the water content and the diffusion of water within the disc, which occur with advancing age or degeneration, can interfere with non-degenerated disc nutrition and promote the degenerative process. This disruption of the non-degenerated discs not only results in deformation when under loading, but also allows loss of disc height, adding more stress to the surrounding annulus and nuclear material to pass through the annulus with subsequent bulging and herniation[18]. Therefore, it has higher risk of bulging or herniation during the transition from healthy resilient discs with high water content to a relatively dry and scarred discs[19]. This is mirrored in the results of the present study. The prevalence of non-degenerated discs of the non-bulging/herniated group was 74.1%, but the prevalence of mildly degenerated and severely degenerated discs of the non-bulging/herniated group was 22.2% and 3.8%, respectively. Nevertheless, mildly degenerated discs had the highest prevalence of bulging discs (45.8%, 49 of 107 discs). Especially for non-degeneration, mild and severe degeneration in the herniated group, the prevalence rates were 28.6%, 17.1% and 54.3%, respectively (***Fig. 3***).

In this investigation, most bulging and herniated lumbar discs were at the caudal level, while most of the non-bulging/herniated intervertebral discs were located at L1-L2 and L2-L3 (***Fig. 4***), which may be attributed to a heavier mechanical compression at the lumbosacral discs and behavior of the mechanically loaded disc is a complex function of age, water content, and the degree of degeneration[20]. ADC could help investigators determine the possible link between decreased nutrition and degeneration of discs. Linear regression analysis demonstrated a weak inverse correlation between ADC values of NP and lumbar disc level (***Fig. 5***), which showed less evidence in our study than in previous reports. However, the previous results are also contradictory, showing the highest ADC values of non-degenerated discs in either cephalad[11] or caudal discs[7]. Niinimaki *et al*.[12] measured lowest lumbar discs and found no significantly association between anatomic level and ADC values.

The intervertebral lumbar disc matrix consists of a complex network of macromolecules whose composition varies in different regions of the disc[21]-[23]. Modifications of the NP matrix content, especially water and glycosaminoglycan (GAG) contents and disc matrix integrity, with disc degeneration, were reflected in correlated changes in the ADC values. Degeneration results in disorganization and destruction of the collagen network[24]-[26]. Once loss of water content and GAG content of NP occurs and the collagen network has been damaged, the potential feasibility of bulging or herniation may accordingly increase[27],[28]. Thus, the structural composition of the intervertebral disc can be depicted by ADC map[29]. In this study, we compared the mean ADC values in different groups. There was a statistically significant relationship between non-bulging/herniated discs and bulging discs, between non-bulging/herniated discs and herniated discs, but no significant relationship was found between bulging and herniation (*P* > 0.05, ***Fig. 6***). In the current study, we also observed elevated ADC values in six herniated discs with severe degeneration, especially for severe collapse of disc height, that can be explained by liquid freely moving through the cracks and cavitations in the degenerated nucleus pulposus or flowing of water through cracks in the disc structure[11],[20], which may result in no significant difference of mean ADC values between bulging discs and herniated discs.

ADC map has shown the potential to quantitatively evaluate degeneration of disc molecular composition and structural integrity because ADC values are sensitive to water content and arrangement of collagen network structure[9],[30],[31]. This is reflected in the non-bulging/herniated group, as measured by the bulging or herniated groups, which showed significant differences in the mean ADC values between non-degenerated discs and degenerated discs.

Nevertheless, there were some limitations of ADC map. First, echo planar imaging sequence is highly sensitive to artifacts, especially to motion artifacts that include gastrointestinal and respiratory movement. In order to minimize and control the possible effect of these artifacts, we not only required every participant to maintain quiet respiration, but also compressed all the subjects in the lower abdomen with abdominal bandage. Second, flow artifact caused by the cerebrospinal fluid often results in confusion between NP and AF and it is easy to cause a measurement bias. To minimize the bias, we placed ROI in the middle slice of sagittal T_2_WI and copy to the same slice of ADC map.

As a noninvasive method of measuring the diffusion of water within tissue *in vivo*, ADC value is a potentially quantitative tool to assess componential and molecular alterations accompanied with disc abnormalities. Additionally, it may estimate therapeutic effect in which standard MRI does not show any change even though clinical symptoms may improve or deteriorate.
